# Novel roles of cardiac natriuretic peptides in renal compensatory hypertrophy

**DOI:** 10.1186/2050-6511-16-S1-A22

**Published:** 2015-09-02

**Authors:** Andrea Schreiber, Janina Staffel, Daniela Valletta, Michaela Kuhn, Frank Schweda

**Affiliations:** 1 Institute of Physiology, University of Regensburg, Germany; 2 Institute of Physiology, University of Würzburg, Germany

## Background

Loss of renal tissue and renal function results in an increase in function and mass of the remaining intact kidney tissue. A prominent example for this so called renal compensatory hypertrophy is the removal of one kidney (uninephrectomy, UNx) for instance in living kidney donors. Although it is well established that kidney size and function of the remaining kidney markedly increase in these patients and that these adaptations are a prerequisite for living kidney donation, several open questions regarding the regulation of the compensatory mechanisms remain. For instance the initial signals inducing the increase in glomerular filtration rate (GFR) are largely unknown. Based on previous studies demonstrating that I. the rapid increase in GFR post-UNx is mediated by a circulating factor and II. the cardiac natriuretic peptides ANP and BNP both are capable of increasing the GFR, we speculated that natriuretic peptides might be the long sought factor mediating the functional adaptation of kidney function in response to a loss of kidney tissue.

## Results

Our observations in different gene targeted mouse models reveal that natriuretic peptide signaling via guanylyl cyclase-A is critical for the rapid increase in GFR which occurs within the first days post uninephrectomy. Thereafter, the functional adaptation and the hypertrophy of the remaining kidney is independent of the cardiac natriuretic peptides, so that GFR and kidney size are regularly elevated six weeks post-UNx even in the absence of GC-A. However, natriuretic peptide / GC-A signaling has a marked renoprotective effect in this chronic phase of renal compensatory hypertrophy, since it prevents podocyte damage and albuminuria. These beneficial effects of GC-A activation on renal integrity is independent of the blood pressure and is mediated via a direct effect on podocytes.

## Conclusion

Natriuretic peptide / GC-A signalling has at least two important functions in the renal adaptation to the loss of kidney tissue: It is critical for the rapid increase in the glomerular filtration rate in the first days after uninephrectomy and it ameliorates podocyte damage and albuminuria in the chronic phase of renal compensatory hypertrophy.

